# Secondary Metabolites From Halotolerant Plant Growth Promoting Rhizobacteria for Ameliorating Salinity Stress in Plants

**DOI:** 10.3389/fmicb.2020.567768

**Published:** 2020-10-22

**Authors:** Kumari Sunita, Isha Mishra, Jitendra Mishra, Jai Prakash, Naveen Kumar Arora

**Affiliations:** ^1^Department of Botany, Deen Dayal Upadhyay Gorakhpur University, Gorakhpur, India; ^2^Department of Microbiology, School for Environmental Sciences, Babasaheb Bhimrao Ambedkar University, Lucknow, India; ^3^DST-Center for Policy Research, Babasaheb Bhimrao Ambedkar University, Lucknow, India; ^4^Department of Environmental Science, School for Environmental Sciences, Babasaheb Bhimrao Ambedkar University, Lucknow, India

**Keywords:** salinity, sustainable agriculture, secondary metabolites, halotolerant PGPR, exopolysaccharides, bioinoculants

## Abstract

Soil salinization has emerged as one of the prime environmental constraints endangering soil quality and agricultural productivity. Anthropogenic activities coupled with rapid pace of climate change are the key drivers of soil salinity resulting in degradation of agricultural lands. Increasing levels of salt not only impair structure of soil and its microbial activity but also restrict plant growth by causing harmful imbalance and metabolic disorders. Potential of secondary metabolites synthesized by halotolerant plant growth promoting rhizobacteria (HT-PGPR) in the management of salinity stress in crops is gaining importance. A wide array of secondary metabolites such as osmoprotectants/compatible solutes, exopolysaccharides (EPS) and volatile organic compounds (VOCs) from HT-PGPR have been reported to play crucial roles in ameliorating salinity stress in plants and their symbiotic partners. In addition, HT-PGPR and their metabolites also help in prompt buffering of the salt stress and act as biological engineers enhancing the quality and productivity of saline soils. The review documents prominent secondary metabolites from HT-PGPR and their role in modulating responses of plants to salinity stress. The review also highlights the mechanisms involved in the production of secondary metabolites by HT-PGPR in saline conditions. Utilizing the HT-PGPR and their secondary metabolites for the development of novel bioinoculants for the management of saline agro-ecosystems can be an important strategy in the future.

## Introduction

The anthropocene era is facing rigorous environmental stress due to aggravated human activities combined with rapid pace of climate change that is negatively affecting agricultural production and its sustainability. Soil salinity is one of the major global issues which is undermining crop yield and jeopardizing productive capacity of soils (Arora et al., 2018). The declining soil fertility and inadequate crop productivity has raised serious concerns for food security of ever rising human population. Reports claim that soil salinity has affected several countries of the world up till now and is growing at a rate of 1–2% each year (Kasim et al., 2016; Shahid et al., 2018). It has been estimated that over one-third of irrigated land could become barren due to increasing levels of soil salinization (Dodd and Perez-Alfocea, 2012). According to the data released by Food and Agriculture Organization (FAO) salinity together with sodicity has affected around 831 million hectares (397 million-hectare saline soils and 434 million hectare sodic soils) of global lands^1^. Soil salinity is mainly prevalent in arid and semi-arid areas of the world where precipitation is insufficient and evapotranspiration rate is higher, leading to water stress conditions and mineral leaching from the plant root zone (Zörb et al., 2019). Excessive use of chemicals, such as fertilizers and pesticides, and climate change are also responsible for increasing soil salinity around the globe. Salt affected soils are characterized by the high electrical conductivity of soil saturation extract (ECe) i.e., more than 4 dS/m and contain excess amounts of salts of bicarbonates, chlorides, and sulfates of Na^+^ (sodium ion), Ca^2+^ (calcium ion), and Mg^2+^ (magnesium ion) which impairs plant health (Rengasamy, 2006; Numan et al., 2018). Such soils are poor in nutrient content, microbial activity, biomass, and organic matter and thus are rendered as infertile (Diacono and Montemurro, 2015). Salinization also interferes with nutrient assimilation, creates ionic disequilibrium due to accumulation of Na^+^ and Cl^–^ in their cells, generates reactive oxygen species (ROS), perturbs carbon and nitrogen metabolism, and reduces rate of photosynthesis in stressed plants (Kang et al., 2019). Soil salinization significantly impacts the economy and as per a study by Qadir et al. (2014), salt-induced land degradation of irrigated areas may cost up to US$ 27.3 billion in a year just because of the loss in crop production. Reclamation of such salt degraded lands is of utmost importance in order to meet the future food demands of predicted human population of 10 billion in 2050 (Egamberdieva et al., 2019).

**FIGURE 1:**
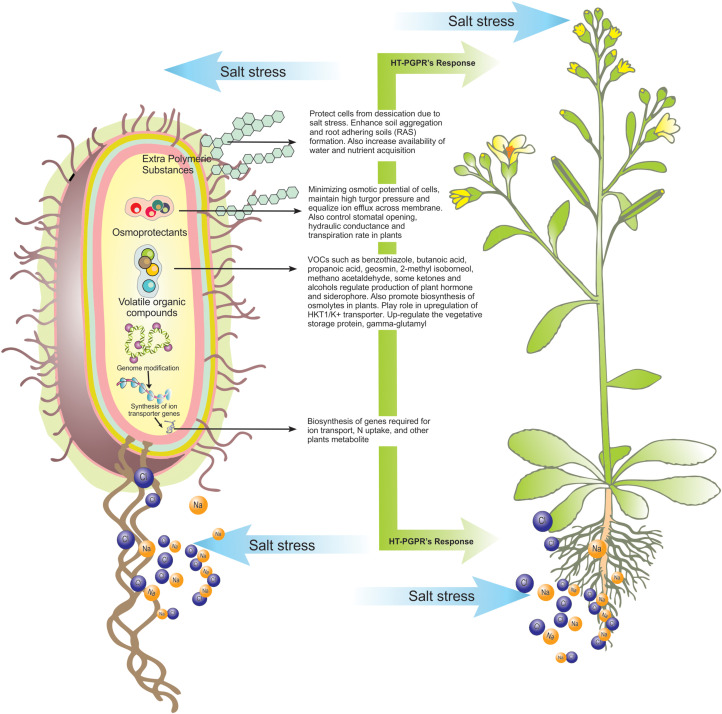
An overview of possible roles of secondary metabolites produced by HT-PGPR in plant’s health improvement under salt-stress.

Physical and chemical remediation of saline soils i.e., soil leaching, flushing, gypsum, and lime treatment are time-consuming and cause loss of biodiversity of indigenous species of plants and microbes (Egamberdieva et al., 2019). To circumvent these constraints, we need to find sustainable methods which are cheaper and eco-friendly in origin, to restore saline degraded lands. Recent studies have shown the potential of halotolerant plant growth promoting rhizobacteria (HT-PGPR) and their secondary metabolites in amelioration of salinity stress in affected plants/crops. HT-PGPR are capable of producing a diverse array of secondary metabolites for protection (of both the host bacterial cells and symbiotic plant) and plant growth promotion even under salinity stress (Abbas et al., 2019). Apart from producing phytohormones, siderophores, solubilization of nutrients such as phosphorous (P), zinc (Zn), potassium (K), and 1-aminocyclopropane-1-carboxylic acid (ACC) deaminase to lower stress ethylene level, HT-PGPR have been reported to produce certain metabolites playing a direct role in salt tolerance and these include osmoprotectants (Upadhyay and Singh, 2015), exopolysaccharides (EPS) (Ashraf et al., 2004) and volatile organic compounds (VOCs) (Vaishnav et al., 2015). Many of these metabolites are exclusively produced during abiotic stress conditions and help in the plant’s survival under adverse environmental conditions (Figure 1). These compounds also assure the ecological fitness of plants by maintaining ionic equilibrium through Na^+^/K^+^ transporter, improving water potential, and expressing salt overly sensitive (SOS) genes involved in stress tolerance under saline conditions (Bharti et al., 2016; Liu et al., 2017a). The review highlights the potent roles of HT-PGPR and their secondary metabolites which hold a promising future for use as next-generation bioinoculants for salt-affected agro-ecosystems.

## HT-PGPR: Diversity and Their Impact on Crop Improvement

Halotolerant plant growth promoting rhizobacteria are now well known for their capability to tolerate and mitigate salinity stress in plants. Several species of HT-PGPR such as *Arhrobacter, Azospirillum, Alcaligenes, Bacillus, Burkholderia, Enterobacter, Microbacterium, Klebsiella, Pseudomonas, Streptomyces, Rhizobium* and *Pantoea* have been reported to alleviate salt stress in crops (Abbas et al., 2019; Arora et al., 2020). In a study by Nadeem et al. (2013), inoculation of wheat by HT-PGPR *Pseudomonas putida*, *Enterobacter cloacae*, *Serratia ficaria*, and *Pseudomonas fluorescens* sown in naturally saline fields (ECe = 15 dS m^–1^) significantly enhanced germination percentage, germination rate and index of wheat seeds by 43, 51, and 123%, respectively, as compared to untreated control. These strains also significantly enhanced yield of wheat in saline field. In another study, inoculation of salt tolerant *Pseudomonas argentinensis* HMM57 and *Pseudomonas azotoformans* JMM15 in *Brassica juncea* grown under salinity stress (ECe = 12 dS m^–1^) resulted in marked increase in root, shoot and plant dry weight (Phour and Sindhu, 2020). Aslam and Ali (2018) showed that a halotolerant *Staphylococcus jettensis* F-11 showed threefold increase in dry weight mass of *Zea mays* under 200 mM salinity stress. Ramadoss et al. (2013) proved that wheat inoculated with *Hallobacillus* sp. SL3 and *Bacillus halodenitrificans* PU62 resulted in increased root length by 90% and dry weight by 17.4% under 320 mM NaCl stress. Several examples of HT-PGPR are now known and they are a very diverse group with some important examples quoted in Table 1. Although it is understood that HT-PGPR can help in alleviating salinity stress and enhance crop productivity (in salt affected soil), there still is a lot to explore regarding the interactions and mechanisms occurring between these microbes and plants during a multi-dimensional stress such as salinity. Roles of secondary metabolites produced by HT-PGPR are crucial and have been explained in the subsequent sections.

**TABLE 1:** Secondary metabolites produced by HT-PGPR and their impact on salt tolerance in plants.

Name of the HT-PGPR	Salt (NaCl) concentrations	Host plant	Type of Secondary metabolite(s)	Effect on the plant’s health	Types of study	References
*Paenibacillus yonginensis* DCY84T	300 mM NaCl	Ginseng (*Panax ginseng* L.)	Osmoprotective compounds	Enhanced nutrient availability, induction of defense-related systems ion transport, antioxidant enzymes, total sugars, ABA and root hair formation	Pot	Sukweenadhi et al., 2018
*Bacillus mojavensis* S1 and *Pseudomonas fluorescens* S3	200 mM NaCl	Barley (*Hordeum vulgare* L.)	Osmoprotective compounds	Alleviation of Na+ concentration in plants Stimulation of root development, enhanced water and nutrient uptake	Pot	Mahmoud et al., 2020
*Pseudomonas* sp. UW4	800 mM NaCl	Tomato (*Solanum lycopersicum* L.)	Osmoprotective compounds	Increased root and shoot length, total dry weight and chlorophyll content	Pot	Orozco-Mosqueda et al., 2019
*Rhizobium* sp. IC3123	16 mM NaCl	Pigeon pea (*Cajanus cajan* L)	EPS	Enhanced germination percentage, pod number, seed yield, protein content and nodule formation (per plant)	Pot & field	Tewari and Sharma, 2020
*Leclercia adecarboxylata* MO1	120 mM NaCl	Tomato (*S. lycopersicum* L.)	VOCs	Enhancment in soluble sugars, i.e., glucose, sucrose, fructose, and organic acids, i.e., citric acid, malic acid, amino acids, i.e., serine, glycine, methionine, threonine, and proline in the fruits	Pot	Kang et al., 2019
*Arthrobacter woluwensis* (AK1), *Microbacterium oxydans* (AK2), *Arthrobacter aurescens* (AK3), *Bacillus megaterium* (AK4), and *Bacillus aryabhattai* (AK5)	200 mM NaCl	Soybean (*Glycine max* L.)	VOCs	Increased level of antioxidant enzymes and K+ uptake; reduced Na+ ion concentration in plant tissue	Pot	Khan et al., 2019
*Pseudomonas aeruginosa* PF23	500 mM NaCl	Sunflower (*Helianthus annuus* L.)	EPS	Production of salicylic acid (SA), enhancement in plant growth parameters and reduction in disease incidence	Field	Tewari and Arora, 2018
*Brevibacterium iodinum* RS16, *Micrococcus yunnanensis* RS222, *B. aryabhattai* RS341, *Bacillus licheniformis* RS656	100 mM NaCl	Canola (*Brassica napus* L.)	EPS	Increased vigor index, fresh weight and growth hormones; production of stress alleviating enzymes	Pot	Hong et al., 2017
*Pseudomonas isolate* P17	125 mM NaCl	Sunflower (*H. annuus* L.)	EPS	Plant growth promotion and biocontrol against phytopathogenic fungus	Pot & field	Tewari and Arora, 2016
*Paraburkholderia phytofirmans* PsJN	150–200 mM NaCl	Thale cress (*Arabidopsis thaliana* L.)	VOCs	Improved plant growth	Pot	Ledger et al., 2016
*Klebsiella*, *Pseudomonas*, *Agrobacterium*, and *Ochrobactrum*	100 mM NaCl	Peanut (*Arachis hypogaea* L.)	VOCs	Nutrients uptake, ion homeostasis, and defense against ROS	Pot	Sharma et al., 2016
*P. fluorescens* 002	150 mM NaCl	Maine (*Zea mays* L.)	VOCs	Improved plant growth	Pot and field	Zerrouk et al., 2016
*Bacillus amyloliquefaciens* SQR9	100 mM NaCl	Maize (*Z. mays* L.)	VOCs	Increase in chlorophyll and total soluble sugar contents, improved K+/Na+ ratio and antioxidant enzymes production	Pot	Chen et al., 2016
*Enterobacter* sp. MN17 and *Bacillus* sp. MN54	400 mM NaCl	Quinoa (*Chenopodium quinoa* L.)	EPS	Improved plant-water relationship	Pot	Yang et al., 2016
*Pseudomonas simiae* AU	100 mM NaCl	Soybean (*G. max* L.)	VOCs	Decrease root Na+ accumulation and increase in proline and chlorophyll content	Pot	Vaishnav et al., 2016
*Bacillus pumilus* (STR2), *Halomonas desiderata* (STR8), and *Exiguobacterium oxidotolerans* (STR36)	500 mM NaCl	Maize (*Z. mays* L.)	EPS and osmoprotective compounds	Improved plant growth, influenced indigenous microbial communities	Pot	Bharti et al., 2015
*Bacillus subtilis* strain GB03	150 mM NaCl	White clover (*Trifolium repens* L.)	VOCs	Decreased Na+ accumulation, increase in chlorophyll content, leaf osmotic potential, cell membrane integrity	Pot	Han et al., 2014
*Enterobacter* sp. EJ01	200 mM NaCl	*A. thaliana* and tomato (*S. lycopersicum)*	VOCs	Enhanced plant growth and increase in salt-stress tolerance	Pot	Kim et al., 2014
*B. pumilus* STR2, *Halomonas desiderata* STR8, and *Exiguobacterium oxidotolerans* STR36	500 mM NaCl	Mint (*Mentha arvensis* L.)	EPS	Improved nutrient uptake and antioxidant machinery	Pot	Bharti et al., 2014
*Halomonas variabilis* HT1 and *Planococcus rifietoensis* RT4	200 mM NaCl	Chickpea (*Cicer arietinum*)	EPS	Increased plant growth, improved soil nutrient status	Pot	Qurashi and Sabri, 2012

## Major Secondary Metabolites of HT-PGPR and Their Role in Salt-Stress Alleviation in Plants

There are several mechanisms by which halotolerant rhizobacteria provide resilience to plants under saline conditions. However, production of secondary metabolites such as EPS, VOCs and compatible solutes (proline, trehalose, and glycine betaines, etc.) by HT-PGPR have been found to directly modulate plant’s cellular responses through regulation of *SOS1* gene (Bharti et al., 2016); expression of stress regulating genes (Morris and González, 2009); expression of high-affinity K^+^-transporter (HKT1) genes (Kasotia et al., 2016); genes for antioxidant protein and ethylene biosynthesis (Kwon et al., 2010) involved in the alleviation of salt-stress. Major secondary metabolites produced by HT-PGPR and their role in mitigation of salt stress in plants are mentioned in Table 1. In the following section, role of key metabolites, produced or supported by HT-PGPR, in salt-tolerance and its mitigation have been discussed.

### Osmoprotectants/Compatible Solutes

**FIGURE 2:**
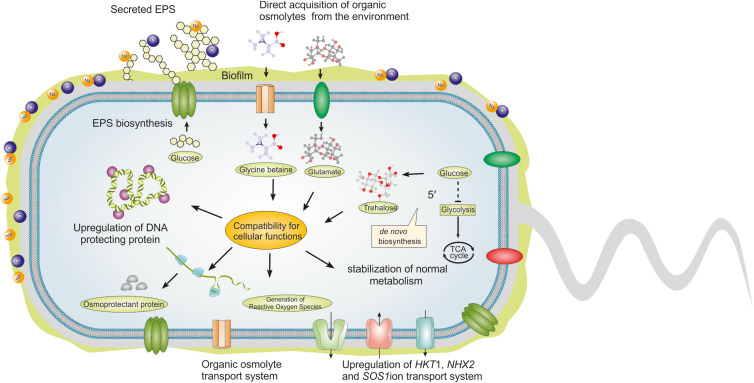
Role of osmoprotective compounds and exopolysaccharides (EPS) produced by HT-PGPR in salt-tolerance. Under salt stress, HT-PGPR produce osmoprotective compounds and EPS. Osmoprotective compounds or compatible solutes can be accumulated either through direct acquisition from the surrounding environment (if available) or through *de novo* biosynthesis. In general, osmoprotective compounds are highly soluble and carry no net charge at physiological pH. Accumulation of osmoprotective compounds at high intracellular concentrations don’t interrupt vital cellular processes. The utmost importance of osmoprotective compounds is to ensure cellular compatibility for the normal metabolic process. Apart from these, they also take part in the upregulation of ion transporters essential for restoring the osmotic equilibrium. Production of EPS by HT-PGPR is strongly correlated with the plant’s survival in high salt concentrations. HT-PGPR can secrete EPS or form biofilm under salt stress. Physiological and chemical properties of the biofilm are also linked with enhancing water retention and neutralizing the harmful effect of salts. The anionic EPS can hold several times its weight of water and at the same time binds with positive salt ions.

Water homeostasis is vital for the proper functioning of physiological and metabolic processes in plants. Accumulation of excess salt in soil perturbs water uptake by plant cells and creates osmotic stress and ionic toxicity (accumulation of Na^+^ and Cl^–^) that in turn inhibits growth and developmental process (Carmen and Roberto, 2011). The hypertonic conditions and abundance of Na^+^ and Cl^–^ ions disturb essential physiological activities that include root and stem growth, maturation of cell structure, transpiration and photosynthesis, enzyme activities, nutrient uptake, hormonal status and many more (Munns, 2002; Pérez-López et al., 2012). To acclimatize under saline conditions, HT-PGPR produce low molecular weight metabolites, known as osmoprotectant compounds or compatible solutes that help in minimizing osmotic stress, maintain high turgor pressure, and equalize ion efflux across the plasma membrane in their mutualistic partner plants (Dodd and Perez-Alfocea, 2012). These compounds also control stomatal opening, hydraulic conductance, and transpiration rate to alleviate the water deficiency in plants (Paul and Lade, 2014; Saghafi et al., 2019). Amino acids and their derivatives (e.g., proline, glutamate, glycine betaine and ectoine), polyols (e.g., glycerol, inositol, sorbitol and mannitol), and non-reducing sugars (e.g., trehaloses) are some of the major compatible solutes produced by HT-PGPR under saline conditions (Sleator and Hill, 2002). Bacteria acquire these compounds either from the surrounding environment or synthesize them *de novo* in response to salt stress and help in amelioration of this abiotic stress in plants through mechanisms as shown in Figure 2. It is a well-known fact that under osmotic stress synthesis of proline gets accelerated in plant cells as compared to normal conditions (Kaur and Asthir, 2015). It is also well documented that HT-PGPR can enhance proline production in plants under salinity stress (Abbas et al., 2019). In response to different environmental stresses such as drought, high salinity, and heavy metals, proline can act as reactive oxygen species (ROS) scavenger, regulate cytosolic acidity, or stabilize the structure of proteins (Krasensky and Jonak, 2012). Biosynthesis of proline involves combined action of enzymes γ-glutamyl kinase, γ-glutamyl phosphate reductase, and δ1-pyrroline-5-carboxylate reductase for the majority of bacteria. This catalytic reaction is stimulated by genes *proB, proA*, and *proC* (Kunst et al., 1997; Roy and Hill, 2002). Nitrogen-fixing bacteria have been reported to show elevated proline metabolism due to increased activity of proline dehydrogenase (PDH) enzyme during salinity stress (Kohl et al., 1994; Meena et al., 2019). A HT-PGPR, *Bacillus fortis* SSB21, was reported to increase the levels of proline along with a reduction in lipid peroxidation and ROS, alongwith upregulation of stress regulating genes *CAPIP2, CaKR1, CaOSM1*, and *CAChi2* in capsicum under saline conditions (1 and 2 g NaCl kg^–1^ soil) (Yasin et al., 2018). Bharti et al. (2014) reported that *Mentha arvensis* inoculated with HT-PGPR belonging to genus *Bacillus, Halomonas*, and *Exiguobacterium* showed a higher foliar proline content as compared to control plants. Pan et al. (2019) illustrated that inoculation of HT-PGPR plays an important role in maintaining ion homeostasis (K^+^/Na^+^) in salt-sensitive plants and helps in accumulation of osmolytes including soluble sugars and proteins as well as increased bioavailability of nutrients like N, P, K, Ca, and Mg, imparting tolerance against adverse effects of salinity. Inoculation of salt tolerant *Paenibacillus yonginensis* DCY84^*T*–^ on ginseng seeds resulted in the accumulation of total soluble sugars, proline, and polyamine content under 300 mM salt stress. The treatment also enhanced nutrient availability, chlorophyll content, abscisic acid (ABA) biosynthesis, and induction of stress-responsive genes in plants under salt stress (Sukweenadhi et al., 2018). Glycine betaine (GB) is another class of compatible solute known to reciprocate salinity induced stresses in plants by accumulating in the cytosol, which in turn reduces the osmotic stress thereby maintaining the overall integrity of the plant cell (Hoque et al., 2007; Wutipraditkul et al., 2015). These are nitrogenous compounds that exist in the zwitterionic state and are known to naturally accumulate during environmental stress conditions (Giri, 2011). Malekzadeh (2015) showed that GB when applied exogenously improved salt-tolerance in soybean which was evident by decreased Na^+^ concentration and increased superoxide dismutase (SOD) and catalase (CAT) activity in treated plants. HT-PGPR are capable of inducing the synthesis and accumulation of GB in salt stressed plants and hence it is better to utilize them for this purpose. Studies reveal that GB synthesis in *Bacillus subtilis* is triggered by the action of two enzymes namely (i) type III alcohol dehydrogenase which oxidizes choline, a precursor molecule to the intermediate compound glycine betaine aldehyde and (ii) a glycine betaine aldehyde dehydrogenase which forms the final product (GB) (Kappes et al., 1999; Roy and Hill, 2002). A GB biosynthetic gene, *codA* (for enzyme choline oxidase) from *Arthrobacter globiformis*, which converts choline into GB, has been widely used for GB production in transgenic plants (Giri, 2011). Inoculation of HT-PGPR *Bacillus* HL3RS14 increased levels of GB in maize and also promoted growth of plants under salinity stress (Mukhtar et al., 2019). In the same way, *B. subtilis* BERA71 inoculated plants showed higher content of GB and other osmolytes in *Acacia gerrardii* under saline conditions (Hashem et al., 2016). Ectoine (1,4,5,6-tetrahydro-2-methyl-4-pyrimidinecarboxylic acid) is another osmolyte that gets accumulated in plant cytoplasm on the exposure of salinity stress (Masouleh, 2019). Synthesis of ectoine in bacteria involves role of three enzymes i.e., L-2,4-diaminobutyric acid aminotransferase, L-2,4-diaminobutyric acid acetyl transferase and L-ectoine synthase (triggered by genes ectB, ectA, and ectC respectively) to compensate nitrate impairment in plant roots (Moghaieb et al., 2006, 2011). In HT-PGPR, the amount of ectoine formation is directly proportional to the increase in osmotic pressure on the cell due to different reasons including salinity stress (Grammann et al., 2002). In a study, ectoine extracted from a halophilic *Chromohalobacter salexigens* KT989776 was reported to enhance seed germination of flax as well as reduced sodium accumulation, peroxidase and phenoloxidase activity in plants (Elsakhawy et al., 2019). Another osmoprotectant utilized by HT-PGPR are trehaloses, which are non-reducing disaccharides in which two glucose moieties are linked by α,α-(1,1)-glycosidic bonds (Chandra et al., 2011). Plants lack the machinery to produce trehalose, but HT-PGPR play important role by producing this osmoprotectant and helping plants under salinity stress. There are enzymes such as trehalose synthase, alpha-trehalose-phosphate synthase, and trehalose-6-phosphate phosphatase (coded by *otsAB* genes) which are involved in the formation of trehaloses in HT-PGPR (Vílchez et al., 2016). Research has revealed that most of the bacteria follow two routes i.e., treY/treZ and treS, for trehalose synthesis and to withstand the harsh effects of salinity stress. Like many other osmoprotectants, it has been observed that the levels of trehaloses spike during drought and salinity stress (Duan et al., 2009). Rodríguez-Salazar et al. (2009) showed that inoculation of *Azospirillum brasilence* overexpressing trehalose biosynthesis gene improved leaf and root biomass of maize plants and also imparted osmotic stress tolerance to them. *Phaseolus vulagris* when inoculated with *Rhizobium etli* having trehalose-6-phosphate synthase overexpressing gene, showed adaptation to osmotic stress, enhanced number of nodules as well as resulted in higher plant biomass (Suárez et al., 2008).

Osmolyte production and accumulation by HT-PGPR is thus one of the key mechanisms which helps in minimizing various stresses caused by salinity in the plants. Genetic manipulation of secondary metabolite producing genes from HT-PGPR can also be utilized for designing salt-resilient transgenic crops in order to reduce agricultural losses (Rodríguez-Salazar et al., 2009). However, ample research regarding salt stress responses in HT-PGPR is required for a better understanding of the metabolic pathways involved in their production. This can give new insights for the development of innovative and novel biotechnological products for enhancing crop productivity of saline agro-ecosystems.

### Exopolysaccharides (EPS)

Exopolysaccharides are commonly produced metabolites of HT-PGPR. Under extreme saline conditions about 40–90% of the extracellular matrix of bacterial weight is due to EPS formation only (Flemming and Wingender, 2001; Fatima and Arora, 2019). EPS help in microbial attachment to plant roots, in biofilm formation to protect cells from desiccation due to salt stress and enhance mobility of root associated bacteria (Ruppel et al., 2013; Qin et al., 2016; Liu et al., 2017b). Production of EPS may vary with bacterial growth phase, medium composition, and exposure of environmental stressors such as salinity and drought (De Vuyst and Degeest, 1999; Kaushal and Wani, 2016). EPS have also been reported to exhibit high antioxidant property and confer tolerance to bacteria against ROS- dependent cell death. In this context, Meneses et al. (2017) demonstrated EPS-mediated protection of endophytic bacterium *Gluconacetobacter diazotrophicus* against oxidative damage *in vitro* and during colonization of rice plants. Similarly, EPS produced by a halotolerant endophyte *Glutamicibacter halophytocola* KLBMP 5180 was evaluated for its multilayer antioxidant activity that can be exploited to alleviate salt stress damage in crops (Xiong et al., 2020). EPS are also supposed to be involved in soil aggregation and increasing root adhering of soil (RAS) by the formation of a sheath around plant’s roots thereby increasing availability of water and acquisition of nutrients such as N, P, K and Fe from the soil (Gupta et al., 2015; Etesami and Beattie, 2018). Mukherjee et al. (2019) reported that under salt stress conditions EPS produced by *Halomonas* sp. EX01 helped in osmotic stress tolerance and promotion of rice growth. EPS can reduce ionic toxicity in plants by minimizing Na^+^ influx through an expression of HKT1/K^+^ transporter (Zhang et al., 2008). By influencing key metabolic processes in plants and retaining physicochemical properties of soil, EPS producing bacteria can also contribute to the improvement of crop productivity under saline conditions (Qurashi and Sabri, 2012; Xiong et al., 2019). Apart from these defined roles, bacterial EPS are also linked with cellular sensing and recognition process in the rhizosphere, protection of plant from phytopathogens and to serve as a carbon source during nutrient deficient conditions (Tewari and Arora, 2016; Mishra and Arora, 2018). Tewari and Arora (2018) reported that inoculation of EPS producing *Pseudomonas* sp. enhanced the yield of sunflower crop in a highly saline field (EC > 10 dS/m). The study also reported that the inoculation of HT-PGPR also reduced incidence of charcoal rot disease in *Macrophomina phaseolina* infested saline soil. Earlier, Ashraf et al. (2004) demonstrated the role of EPS producing salt-tolerant *Bacillus amyloliquefaciens, Bacillus insolitus, Microbacterium* spp., and *Pseudomonas syringae* in improving the growth of wheat by the inhibition of Na^+^ influx into stele of plant growing under salinity stress. Similarly, Mahmood et al. (2016) concluded that under salinity stress, treatment of mung bean with EPS producing *Enterobacter cloacae* and *Bacillus drentensis* enhanced nutrient availability and water uptake in plants by the formation of biofilm in the root zone. In another study, inoculation of EPS producing endophyte, *Pantoea alhagi* NX-11 improved salt tolerance of rice seedlings by improving the antioxidant activity resulting in better growth in comparison to the plants treated with EPS mutant NX-11^*eps*–^ (Sun et al., 2020). Likewise, treatment of maize with EPS producing *Azotobacter chrococcum* strains C5 and C9 under saline conditions alleviated the saline stress by several means i.e., increasing K^+^/Na^+^ ratio and ions uptake (Na^+^, K^+^, Ca^2+^, Mg^2+^), chlorophyll content, and accumulation of proline and polyphenols (Rojas-Tapias et al., 2012). EPS based bioformulation of *Alcaligenes* sp. was found to be very effective in enhnacing growth and reducing osmotic stress in rice when grown in saline conditions (Fatima et al., 2020). Overall, research suggests that EPS produced by PGPR play an important role to overcome salinity stress in plants and can be used in bioinoculants to improve the rhizosphere colonization, soil quality, and nutrient acquisition in saline conditions. EPS can also be used as amendments in bioinoculants for protecting the PGPR microbe from initial stress faced in saline soils at the time of inoculation.

### Volatile Organic Compounds (VOCs)

Halotolerant plant growth promoting rhizobacteria when exposed to stress conditions like salinity, are known to produce VOCs which are low molecular weight compounds (below 300 Da), and lipophilic in nature, with low boiling point (Vespermann et al., 2007; Kanchiswamy et al., 2015). A vast array of microbial volatiles have been reported and among them, many are recognized for their potential in improving overall plant health (Kanchiswamy et al., 2015). These compounds are often used as markers for specific detection of microbial species in the environment and in accessing the nature of interaction among microbial communities (Fiedler et al., 2001; Tirranen and Gitelson, 2006). Their roles in regulating bacterial motility in plant-microbial interactions, modulating virulence factors, biosynthesis of osmolytes like GB, phytohormones (auxins, cytokinins, and gibberellins) and siderophores are also studied and reported (Ryu et al., 2004; Liu and Zhang, 2015; Sharifi and Ryu, 2018). In a study, Tahir et al. (2017) showed that VOCs albuterol and 1,3-propanediol produced by *B. subtilis* SYST2 modulated levels of plant hormones expansin, auxin, gibberellin, cytokinin, and ethylene. Geosmin, dimethyl disulfide, 2,3-butanediol, and acetoin are the most studied bacterial VOCs that help in soil formation processes, composting, sulfur nutrition, auxin homeostasis and cell expansion, induction of systemic and drought tolerance in plants (Stahl and Parkin, 1996; Li et al., 2004; Zhang et al., 2007; Cho et al., 2008; Meldau et al., 2013). In a study, Gutierrez-Luna et al. (2010) reported emission of 22 types of VOCs (aldehydes, ketones, and alcohols were the most abundant) by six *Bacillus* strains and further revealed that most of VOCs can stimulate primary root growth and lateral root development in *Arabidopsis thaliana*. Similarly, volatile compounds produced by root associated *Microbacterium* spp. showed increment in root and shoot biomass of *A. thaliana.* The study revealed that even a brief exposure of bacterial VOCs also stimulated plant growth suggesting that these can be used to prime crops without direct and prolonged exposure of plants to bacteria. Moreover, it was also demonstrated that the VOC mediated plant growth promotion was tissue specific and showed biomass increment only in plants exposed to volatiles via roots (Cordovez et al., 2018). Two bacterial volatiles, 4-nitroguaiacol, and quinoline produced by salt-tolerant *Pseudomonas simiae* were reported to induce soybean growth under 150 mM salt stress (Vaishnav et al., 2016). A VOC producing HT-PGPR *Paraburkholderia phytofirmans* PsJN, was not only reported to help in salt-stress amelioration but also promoted *A. thaliana* growth in very saline conditions (Ledger et al., 2016). VOCs production is also linked to the regulation of HKT1/K^+^ transporter which is responsible for inhibition of Na^+^ influx during salt stress. For example, Zhang et al. (2008), found that a VOC producing *B. subtilis* improved salt-tolerance in *A. thaliana* by downregulating *HKT1* gene expression. Bhattacharyya and Lee (2017) investigated the role of mixtures of three bacterial volatiles produced by *Alcaligenes faecalis* JBCS1294. The study demonstrated that blends of VOCs (butyric acid, propionic acid, and benzoic acid) increased plant growth and induced salt tolerance in *A. thaliana* by modulating its hormonal pathways and ionic transporters. Systematic exploration of microbial VOCs indicates that these compounds could also be involved in novel biological functions and ecological roles that are presently not known (Kanchiswamy et al., 2015). Although it is certain that VOCs play important roles, such as in regulating growth hormones and ion acquisition, there still is a lot more to be explored regarding the direct role of these metabolites and their potential to ameliorate salt stress in plants. A better understanding of microbial VOCs capable of eliminating the negative effects of salt stress in the plant might be employed in the future for the development of a range of novel bioinoculants with great agronomic importance.

## Future

Halotolerant plant growth promoting rhizobacteria are one of the pre-eminent group of microbes that can be utilized in the engineering of the rhizosphere in saline soils to replenish their fertility and increasing crop yield (Stringlis et al., 2018; Arora et al., 2020). It has been established that secondary metabolites produced by HT-PGPR can be very useful to develop novel bioinoculants for the cultivation of crops in saline soil (Arora, 2019). Secondary metabolites, such as EPS from HT-PGPR not only can overcome issues related to shorter shelf-life but also increase the survival of bacterial/fungal strains in the bioinoculants (Arora and Mishra, 2016). Techniques such as metagenomics can be an important tool for identification, designing and reassembling of novel genetic pathways and for the construction of new biosensors in order to produce secondary metabolites from diverse HT-PGPR (de Frias et al., 2018). Based on the results, metagenomic libraries can be constructed related to structural and functional genes for novel and known metabolites. Similarly, Artificial Gene Operon Assembly System (AGOS) has also come up in which a particular metabolite pathway can be optimized and controlled with chemical modifications. This will help in introducing the assembly of artificial gene clusters in native or heterologous hosts and can increase the overexpression of metabolites of interest (Basitta et al., 2017). Bioinformatic tools can be helpful in deciphering distribution and functional traits within bacterial genomes that are involved in rhizosphere colonization under saline conditions (Barea, 2015). Dragon Explorer of Osmoprotection associated Pathways’ (DEOP), a database on osmolytes is already available providing curated information and facilitating deeper insights on osmolytes and their associated pathways in the microbial genome (Bougouffa et al., 2014). The study of plant microbiome and its intricate mechanisms is a crucial strategy to develop designer salt-tolerant crops (Macdonald and Singh, 2014; Rodriguez et al., 2019). This will unravel the hidden untapped arsenal of endophytes associated with halophytic plants and other extreme habitats. Due to the ability to form symbiotic relationship with hosts, salt-tolerant endophytes should be explored in the future (Qin et al., 2018). Stress tolerant genes can also be incorporated in plants through transposon-mediated genetic modification (Arora et al., 2018). Transcriptome analysis has revealed that genes functioning at high salt concentrations from halotolerant bacteria can be used for the development of stress-tolerant plant varieties to expand agricultural productivity (Das et al., 2020). In addition, nanoencapsulation technology has been recently introduced and can be applied for field trials (De Gregorio et al., 2017). The technique can be used to protect the PGPR from sudden environmental stresses, improve their dispersal and will serve in the controlled release of microbes in the field/rhizosphere (Vejan et al., 2016). Further research on the identification and utilization of more such nanoparticles should be made so that these growth enhancers can be used in sustainable agricultural practices. These high throughput protocols will open new gateways for enhanced production of bioactive products from HT-PGPR, reducing the use of agrichemicals (Backer et al., 2018). Research should also be focused on co-cultivation of potent salt-tolerant strains possessing positive synergy for the production of novel metabolites which can work in tandem to tackle diverse problems (Singh et al., 2019). Interlinking these biotechnological processes with agronomy and investigating the diversity and composition of halotolerant microbes will provide improvement in cultivating crops under stressed conditions. The market size of plant growth regulators is currently increasing at the rate of 3.6%^2^ and is projected to enhance even further in coming future. This presents a scenario of global demand for growth enhancers for plants and hence provides a roadmap for the commercialization of microbial products. However, biostimulants involving HT-PGPR and their metabolites are yet to take off and enter the market. Hence development of tailor made bioformulations from these unique PGPR and their metabolites can be crucial to combat salinity and improve the yield of salt affected agro-ecosystems leading to better productivity and sustainability of agro-ecosystems.

## Conclusion

Halotolerant plant growth promoting rhizobacteria act as a promising probiotic for salt-affected plants and in restoring the natural equilibrium of saline soils. The ability of these bacteria to survive in saline conditions makes them an excellent tool in realizing the targets of sustainable agriculture. Information about halotolerant microbiota, their structure and composition are still in its nascent stage. Hence, extensive research is needed to understand the saline mitigation mechanisms which will provide new insights in designing future bioformulations to help remediate salt degraded lands and to achieve targets of food security.

## Author Contributions

NKA conceptualized the idea and approved the final manuscript to be published. All the authors read, discussed as well as equally contributed to the writing of the manuscript.

## Conflict of Interest

The authors declare that the research was conducted in the absence of any commercial or financial relationships that could be construed as a potential conflict of interest.

## Footnotes

1 http://www.fao.org/soils-portal/soil-management/management-of-some-problem-soils/salt-affected-soils/more-information-on-salt-affected-soils/en/

2 https://www.marketresearch.com/MarketsandMarkets-v3719/Plant-Growth-Regulators-Type-Auxins-9511806/
